# A novel double-sheath negative-pressure versus conventional minimally invasive percutaneous nephrolithotomy for large kidney stone

**DOI:** 10.1038/s41598-023-50237-7

**Published:** 2023-12-27

**Authors:** Kuer-Ban Tuoheti, Xing-Huan Wang, Ting Wang, Yong-Zhi Wang, Tong-Zu Liu, Zhong-Hua Wu

**Affiliations:** Department of Urology, Zhongnan Hospital of Wuhan University, Wuhan, China

**Keywords:** Nephrology, Urology

## Abstract

This study aims to evaluate the therapeutic efficacy of a novel double-sheath negative-pressure minimally invasive percutaneous nephrolithotomy (D-mPCNL) compared to conventional minimally invasive percutaneous nephrolithotomy (C-mPCNL) for large kidney stones. A total of 132 patients diagnosed with large kidney stones in our hospital were included in the study. Among them, sixty-eight patients underwent D-mPCNL, while sixty-four underwent C-mPCNL. Parameters such as operative duration, stone-free rate, incidence of postoperative complications, and the need for auxiliary procedures were evaluated between the two groups. Compared to the C-mPCNL group, the D-mPCNL group demonstrated a significantly shorter operative time (41.97 ± 8.24 min vs. 52.30 ± 13.72 min; P < 0.000), lower rates of auxiliary procedures (5.9% vs. 17.2%; P = 0.041), and lower fever rates (2.9% vs. 14.1%; P = 0.021). The group also had a significantly higher primary stone-free rate (85.3% vs. 70.3%; P = 0.038). However, there were no statistically significant advantages in terms of the final stone-free rate, hemoglobin drops, and stone composition in the D-mPCNL group (P > 0.05). D-mPCNL is a novel surgical method that is safe and effective, reducing operative time, improving stone-free efficiency, and decreasing postoperative complications.

## Introduction

Minimally invasive percutaneous nephrolithotomy (mPCNL) has gained favor among urological surgeons due to its potential benefits, including decreased renal parenchyma stress and lower bleeding compared to standard percutaneous nephrolithotomy^1^. Despite these benefits, mPCNL carries risks such as high renal pelvis pressure and postoperative infection^2,3^.

Numerous attempts have been made to overcome the disadvantages of mPCNL^4–8^. While these efforts have led to improvements, they have not fully resolved all the challenges associated with this technique. To address these deficiencies, we developed a novel double-sheath negative-pressure mPCNL (D-mPCNL), consisting of a 20F Y-shaped sheath as the outer sheath and a 16F Y-shaped sheath as the inner sheath^9^.

In this study, we compared D-mPCNL with conventional minimally invasive percutaneous nephrolithotomy (C-mPCNL) to evaluate whether our double-sheath negative-pressure technique represents a significant improvement in treating large kidney stones.

## Methods

A total of 132 patients with significant radiopaque renal calculi who underwent mPCNL between October 2019 and September 2020 were enrolled in this study. This included 68 cases of D-mPCNL and 64 cases of C-mPCNL. All patients underwent preoperative renal ultrasonography (US), plain radiography of the kidney-ureter-bladder (KUB), and non-contrastive abdominal computed tomography (NCCT) to determine stone size and location. The calculi ranged from minor renal pelvic calculi to full-blown staghorn calculi. Inclusion criteria were ages between 18 and 70 and calculi larger than 20 mm. The density of the calculi (measured in Hounsfield units) was calculated.

All operations were performed by skilled surgeons. The patient was placed in a supine position, and a ureteric catheter was inserted transurethrally. Then, the patient was rolled onto their back. Under ultrasonography guidance, the calyx at the stone or optimal lithotriptic location was punctured. In the C-mPCNL group, the tract was dilated with a fascia dilator, and sheathing was performed with a 20F peel-away sheath. An 8/9.8F ureteroscope was used as a mini-nephroscope. Stone fragmentation was performed using a 550-μm holmium-YAG laser with settings of 1.0–2.5 J and 20–35 Hz. The perfusion inflow rate was set at 90–120 ml/min.

**Figure 1:**
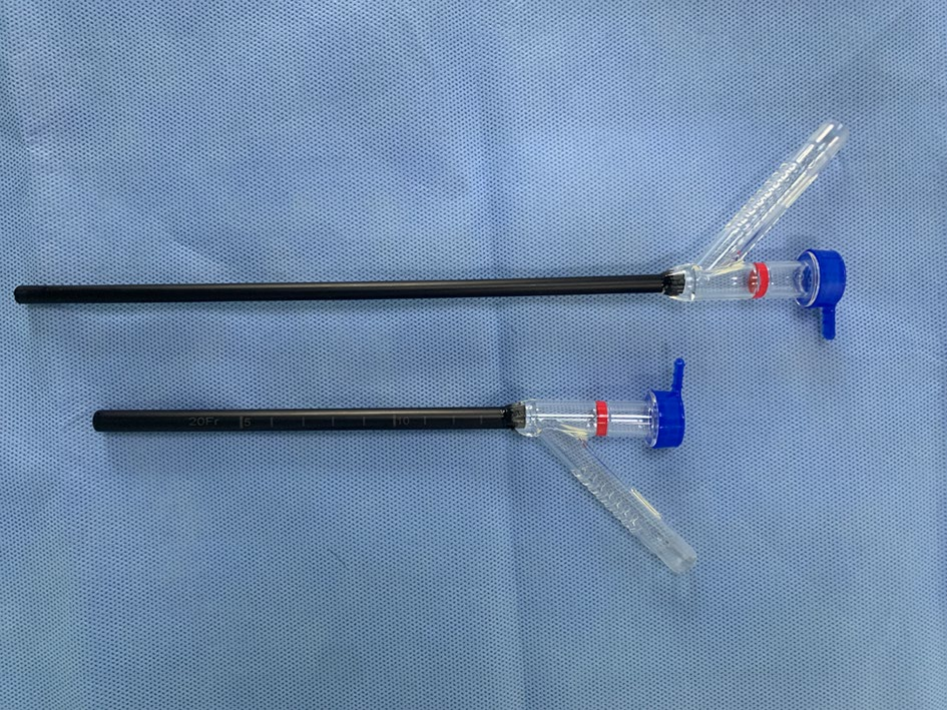
The double-sheath negative-pressure system consists of a 20F Y-shaped outer sheath and a 16F Y-shaped inner sheath.

In the D-mPCNL group, we implemented the double-sheath negative-pressure technology, which consists of an inner and an outer sheath (ClearPetra; Well Lead Medical) (Fig. 1). The outer sheath is a 20F vacuum-assisted Y-type sheath (with a working length of 13 cm) connected to the perfusion flow, and the inner sheath is a 16F vacuum-assisted Y-type sheath (with a working length of 21 cm) connected to the vacuum suction. The gap between the two sheaths serves as the perfusion inflow channel, and the lumen of the inner sheath functions as the perfusion outflow channel. An 8/9.8F ureteroscope was inserted as a mini-nephroscope through the inner sheath, but it was not connected to the irrigation solution.

Lithotripsy was performed using a 550-μm holmium-YAG laser through the ureteroscope's manipulation channel. The parameters were set as follows: energy level of 1.2–2.0 J, frequency of 30–40 Hz, perfusion flow rate of 50–80 ml/min, and vacuum suction pressure of 150–250 mmHg.

**Figure 2:**
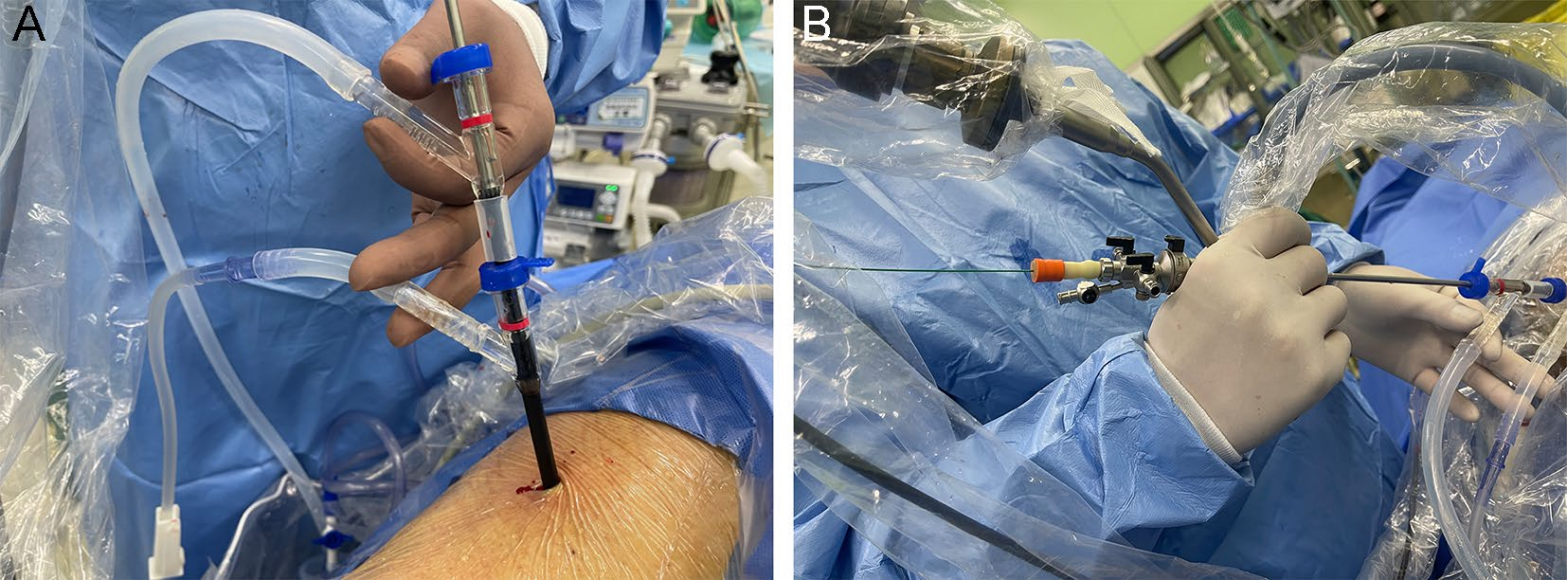
(**A**,**B**) The double-sheath system enables single-handed operation, with the other hand operating the mini-nephroscope.

Clear surgical vision was maintained by aligning the distal end of the inner sheath with that of the outer sheath, while slightly retracting the inner sheath's end to prevent contact with tissue or stones, thus maintaining the perfusion system's patency. To align the sheaths, we either inserted a cut 23 mm segment of the suction tube between the sheaths as a spacer or appropriately trimmed the inner sheath. The sheath’s design, with a pressure vent on the oblique arm of the inner sheath, allowed for fine adjustment of the negative pressure with intermittent thumb presses, achieving a dynamic balance. The inner and outer sheaths' inclined arms could be maneuvered simultaneously with one hand, as depicted in the provided image, allowing precise adjustment of the angle and depth required for the surgery. Since the double sheath system allowed for single-handed operation, with the other hand operating the mini-nephroscope, this setup ensured stable operation and consistent vision (Fig. 2A,B).

**Figure 3:**
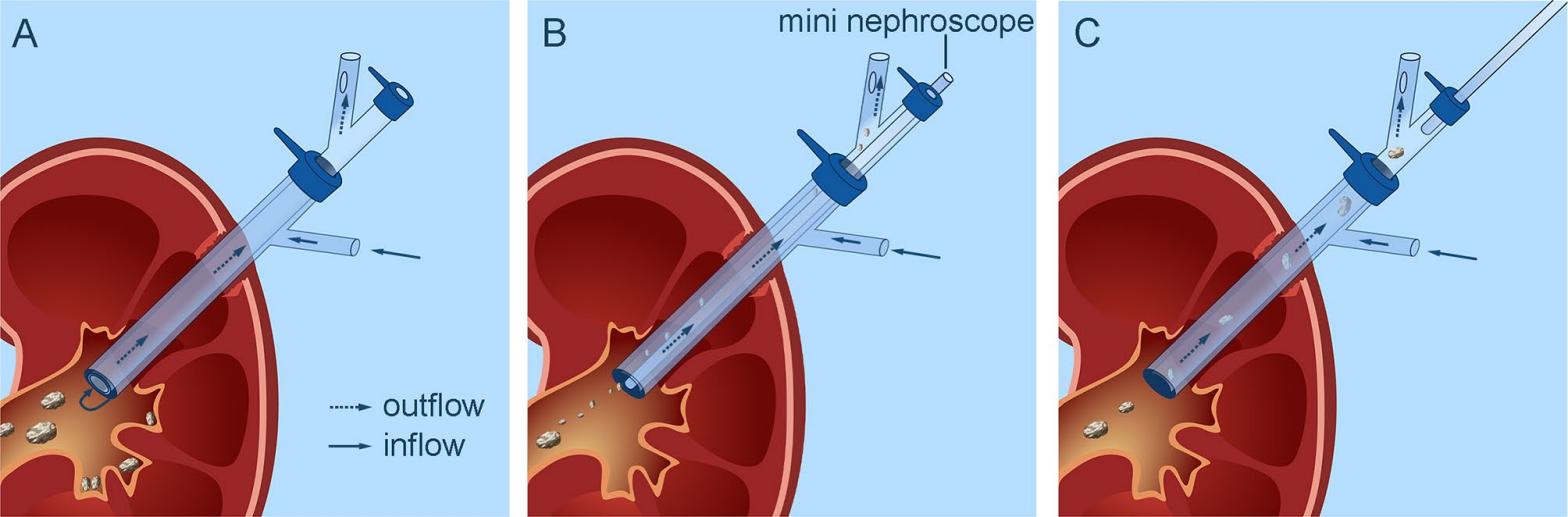
Working Principle of D-mPCNL: (**A**) Establishing a double-sheath negative-pressure system. (**B**) Actively suctioning small stone fragments through the space between the mini-nephroscope and the inner sheath. (**C**) Enhancing the removal of larger stone fragments by withdrawing the mini-nephroscope to the inclined arm of the inner sheath for active suctioning.

Small stone fragments were suctioned out through the gap between the mini-nephroscope and the inner sheath. Slightly larger stone fragments were suctioned out when the mini-nephroscope was returned to the inclined arm of the inner sheath (Fig. 3A–C).

An ultrasonographic check for residual stones was performed before the end of the surgery. Finally, a double-J ureteral stent was placed antegrade into the ureter, and all patients received nephrostomy tubes as standard. The KUB was performed to determine the stone-free rate, with the initial stone-free rate defined as no obvious stones on the first day after surgery and the final stone-free rate determined one month after surgery.

Statistical analyses were performed using SPSS version 25 (IBM Corporation, USA). The mean and standard deviation (SD) were used for quantitative data. The chi-square test (or Fisher's exact test) and independent-sample T-test were used to compare qualitative data for discrete and continuous variables, respectively. P-values < 0.05 were considered statistically significant.

### Ethics approval and consent to participate

This retrospective study was conducted after receiving approval from the Ethics Committee of Zhongnan Hospital of Wuhan University, all procedures were in line with the requirements of the Declaration of Helsinki, and all enrolled patients signed informed consent.

## Results

All procedures were completed safely. Table 1 presents the baseline demographic and clinical characteristics of the D-mPCNL and C-mPCNL groups. There were no significant differences between the groups in terms of age, gender, BMI, or laterality. The size of calculi and stone Hounsfield density were also comparable in both groups (32.49 ± 8.710 mm vs. 33.22 ± 12.22 mm; P = 0.691; 1017 ± 85.12 units vs. 1027 ± 80.62 units; P = 0.458).

**Table 1:** Baseline data of the patients.

Parameters	D-mPCNL group	C-mPCNL group	*P* value
Number of patients	68	64	–
Age (years), (mean ± SD)	44.53 ± 13.76	49.20 ± 13.79	0.054
Gender (male/female)	38/30	35/29	0.890
BMI (kg/m^2^), (mean ± SD)	23.87 ± 2.739	24.50 ± 2.367	0.157
Stone laterality (L/R)	32/36	33/31	0.605
Stone siz (mm), (mean ± SD)	32.49 ± 8.710	33.22 ± 12.22	0.691
Stone-Hounsfield density, ( mean ± SD)	1017 ± 85.12	1027 ± 80.62	0.458

*BMI* body mass index.

Table 2 compares operation-related indices between D-mPCNL and C-mPCNL. There were no statistical differences in hemoglobin drop between the two groups. The D-mPCNL group exhibited a lower fever rate requiring additional intravenous antibiotics compared to the C-mPCNL group (2.9% vs. 14.1%; P = 0.021). Fortunately, other complications such as transfusions (grade II), and interventions (grade III) were not observed in either group. However, auxiliary procedures were required in 4 patients in the D-mPCNL group (2 ESWL, 1 Second-look, and 1 URL) and 11 patients in the C-mPCNL group (6 ESWL, 2 Second-look, and 3 URL) (5.9% vs. 17.2%; P = 0.041). In terms of surgical indices, the D-mPCNL group had a significantly shorter operative time than the C-mPCNL group (41.97 ± 8.24 min vs. 52.30 ± 13.72 min; P < 0.000), and a higher primary stone-free rate (85.3% vs. 70.3%; P = 0.038). However, the final stone-free rates (92.6% vs. 90.6%; P = 0.674) and stone compositions did not show statistically significant differences.

**Table 2:** Treatment outcomes of the two groups.

Parameters	D-mPCNL group	C-mPCNL group	*P* value
Number of patients	68	64	–
Hemoglobin drops (g/L), (mean ± SD)	6.10 ± 5.63	4.89 ± 4.34	0.170
Complications (Clavien grade), n (%)			
Fever (grade II)	2 (2.9%)	9 (14.1%)	0.021
Transfusions (grade II)	0	0	–
Intervention (grade III)	0	0	–
Requiring auxiliary procedure, n (%)	4 (5.9%)	11(17.2%)	0.041
ESWL	2	6	
Second-look	1	2	
URL	1	3	
Operative time (min), (mean ± SD)	41.97 ± 8.24	52.30 ± 13.72	0.000
Primary stone-free rate, n (%)	58 (85.3%)	45 (70.3%)	0.038
Final stone-free rate, n (%)	63 (92.6%)	58 (90.6%)	0.674
Stone composition, n (%)			0.916
Calcium oxalate	54 (79.4%)	50(78.1%)	
Uric acid	8(11.8%)	7(10.9%)	
Carbonate apatite	6 (8.8%)	7(10.9%)	

*URL* ureteroscopy lithotripsy, *ESWL* extracorporeal shock wave lithotripsy.

## Discussion

In recent years, mPCNL has gradually increased, aiming to reduce the risks of bleeding and organ injury associated with the percutaneous access tract^1^. Although mPCNL can achieve comparable stone-free rates to standard PCNL, it has several drawbacks, including difficult fragment removal, longer operative times, and the risk of high renal pelvis pressure leading to infective complications^2,10^.

Some authors have conducted experiments in artificial models to explore the "vacuum cleaner effect" in conventional PCNL. This phenomenon, where kidney stones are passively removed via the irrigation stream, is influenced by various factors such as flush pressure, sheath diameter, and nephroscope design^11^. However, increasing the renal access angle or nephroscope extraction speed can negatively impact the effectiveness of this vortex effect^12^.

PCNL employing lithotripsy instruments with suction capabilities, like EMS LithoClast and Cyberwand, has increasingly been utilized for renal stone treatment, showing promising results^8^. While suction-assisted stone removal can decrease intrarenal pressure (IRP), it does not offer continuous vacuum suction due to interruptions during ultrasonic lithotripsy intervals. The Laser Suction Handpiece, allowing simultaneous laser lithotripsy and suction, requires pulverizing stones into smaller fragments or dust using high-powered lasers. Its current use is mainly in experimental or clinical trial settings, with more extensive research and validation needed to establish its efficacy and safety compared to traditional treatment methods^8,13^.

Negative-pressure access sheath mPCNL treating renal calculi with low IRP is a secure and reliable technique^4–6,14–17^. However, in this system, both the inflow and outflow occur through the same channel. When the nephroscope is withdrawn for larger fragment extraction, the irrigation can neutralize the negative pressure effect, reducing the efficiency of calculi removal and diminishing the low renal pelvic pressure advantage. To compensate for this, our group modified the system with D-mPCNL, where the irrigation inflow channel is the gap between the two sheaths and the irrigation outflow channel is the inner sheath, ensuring independent irrigation inflow and outflow channels throughout the procedure. This provides efficient one-way flow and a semi-closed circuit where irrigation fluid and stone fragments are actively aspirated through the outflow channel.

In our study, compared to the C-mPCNL group, the D-mPCNL group had a shorter operative time (41.97 ± 8.24 min vs. 52.30 ± 13.72 min; P < 0.000), higher primary stone-free rate (85.3% vs. 70.3%; P = 0.038), a lower rate of infective complications (2.9% vs. 14.1%; P = 0.021), and fewer auxiliary procedures (5.9% vs. 17.2%; P = 0.041). These improved outcomes in the D-mPCNL group are likely attributable to the unique design of the double-sheath system, which enhances the efficiency of stone removal while mitigating complications. The double-sheath negative-pressure suction system allows independent channels for inflow and outflow, forming a one-way irrigation fluid that keeps renal pelvic pressure low and reduces the risk of infective complications. Continuous suction provides a clearer visual field, as minor bleeding and calculi dust no longer interfere with vision under high-flow irrigation without increasing renal pelvic pressure.

For calculi dust, the system can directly aspirate it alongside the irrigation flow around the nephroscope during lithotripsy. When the nephroscope is withdrawn to the tail of the inner sheath for larger calculi pieces, the negative-pressure suction effect of the irrigation outflow is no longer offset by nephroscope irrigation. Larger calculi pieces can be actively sucked out, increasing stone extraction efficiency and shortening the operative time, despite the 16F inner sheath being slightly smaller than the 20F sheath used in C-mPCNL. This is particularly significant since the operative time in PCNL often depends more on the time taken to retrieve stone fragments, as stone fragmentation has become less laborious with the advent of high-power laser technology^18^. Furthermore, continuous aspiration can immobilize calculi fragments at the opening of the sheath, resulting in faster and more efficient lithotripsy and reducing the need for manual fragment removal with graspers or baskets.

The current study has several limitations, being susceptible to biases typical of single-center data collected retrospectively. To ascertain whether there are variations in results between D-mPCNL and C-mPCNL, a prospective, randomized trial contrasting the two groups is required. As this study only shows short-term outcomes in a relatively small number of patients, further studies based on longer follow-ups and larger populations are needed to confirm the benefits of D-mPCNL for managing large renal calculi. Incorporating a C-arm at the conclusion of PCNL could reduce residual fragment rates and auxiliary procedures, an aspect we will consider in future studies.

## Conclusions

D-mPCNL is a novel surgical treatment that is safe and effective, reducing operative time, improving stone-free efficiency, and reducing postoperative complications.

## Data Availability

Data supporting the results of this study may be obtained from Professor Zhong-Hua Wu upon reasonable request.

## Abbreviations

D-mPCNL: Double-sheath negative-pressure minimally invasive percutaneous nephrolithotomy
C-mPCNL: Conventional minimally invasive percutaneous nephrolithotomy
KUB: Kidney-ureter-bladder
NCCT: Non-contrastive abdominal computed tomography
BMI: Body mass index
URL: Ureteroscopy lithotripsy
ESWL: Extracorporeal shock wave lithotripsy
IRP: Intrarenal pressure
